# Incidental ablation of ganglionated plexus during atrial fibrillation ablation

**DOI:** 10.1007/s10840-024-01886-9

**Published:** 2024-07-27

**Authors:** Stephen Keane, Darshak Patel, Brian Otto, Lily Englander, Ramanan Kumareswaran, David Lin, Michael P. Riley, Saman Nazarian, Francis E. Marchlinski, Timothy M. Markman

**Affiliations:** 1 Cardiovascular Division, Perelman School of Medicine at the University of Pennsylvania, Philadelphia, PA USA; 2 Abbott Cardiovascular, Plymouth, MN USA

**Keywords:** Atrial fibrillation, Catheter ablation, Cardioneuroablation, Fractionation mapping

## Abstract

**Background:**

Cardioneuroablation targeting the autonomic nerves within ganglionated plexus (GP) has been used to treat atrial fibrillation (AF). Incidental cardioneuroablation may be an important mechanism by which pulmonary vein isolation (PVI) is effective. Automated fractionation mapping software can identify regions of fractionation correlating with GP locations.

**Objective:**

To examine the overlap between standard PVI ablation lesions and fractionated electrograms suggestive of GP.

**Methods:**

We retrospectively examined AF ablations performed from 2021 to 2023 that included only PVI performed using wide antral circumferential isolation without prospective evaluation of fractionation. Retrospectively, a fractionation map was created (width 10 ms, refractory time 30 ms, roving sensitivity 0.1 mv, and threshold of 2). We evaluated the anatomic overlap between PVI lesions and fractionation in regions associated with GP.

**Results:**

Among 52 patients (mean 65 (IQR 46–74) years, 82% male, and 69% paroxysmal AF), sites of fractionation corresponding to GP locations were seen in all cases. PVI ablation incidentally overlapped with fractionation in 50 (96%) patients. On average, 26% of the fractionation corresponding with GP locations were incidentally ablated. The highest proportion of fractionated areas were ablated in the left superior (36%) and right superior (31%) GP regions. More complete incidental ablation of these regions was associated with a greater intraprocedural increase in heart rate (*ρ* = 0.46, *p* < 0.001), which was subsequently associated with freedom from AF during 15.9 ± 5.2 months of follow-up.

**Conclusion:**

Patients undergoing AF ablation universally have fractionated electrograms corresponding to anticipated sites of GP. Partial ablation of these regions frequently occurs incidentally during PVI.

**Graphical Abstract:**

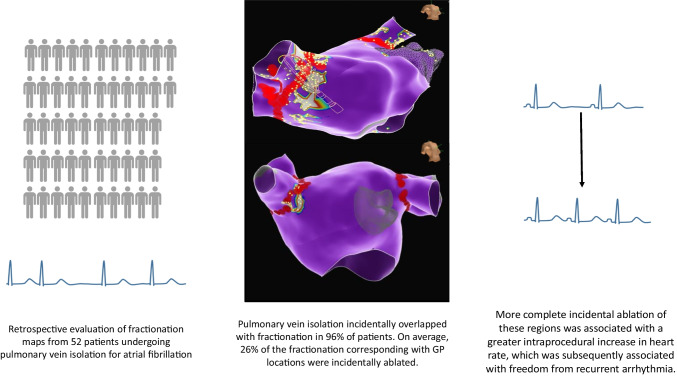

## Introduction

The epicardial surface of the heart contains clusters of autonomic nerves called ganglionated plexus (GP), which are involved in the pathogenesis of cardiac arrhythmias, including atrial fibrillation (AF). The cardiac GP, which includes both parasympathetic and sympathetic nerves, is located within atrial epicardial adipose tissue. GP ablation has been used as a supplementary treatment for AF. Due to anatomic overlap of the intrinsic cardiac autonomic nervous system and typical regions ablated for AF, some degree of atrial denervation can be achieved incidentally during AF ablation [1]. This incidental cardioneuroablation is felt to be a mechanism by which pulmonary vein isolation (PVI) is effective for preventing recurrent AF, including when true isolation of the pulmonary veins is not durably achieved [2–4]. Although incidental denervation has been reported, there is limited data on the variable overlap between routine PVI lesion sets and GPs.

Various methods including high-frequency stimulation, electrogram (EGM) characterization, and an anatomic approach have commonly been used to locate GPs during electrophysiological studies [5, 6]. However, there is currently no consensus on the most effective technique. Automated fractionation mapping software has been developed that can identify regions of fractionation (Ensite X, Abbott). Utilizing a combination of customizable criteria, this system has been shown to identify regions of fractionation correlating with GP locations [7, 8].

Our study aimed to examine the incidental overlap of anticipated GPs based on fractionation and routine PVI lesion sets.

## Methods

We retrospectively examined first time AF ablations performed in the Hospital of the University of Pennsylvania from June 2021 to March 2023. We included patients who underwent PVI only using Ensite X mapping system and excluded patients who had additional left atrial ablation. All procedures were performed under general anesthesia. No patient underwent intentional cardioneuroablation or prospective evaluation of fractionation mapping results during the procedure. Left atrial electroanatomic maps were performed during sinus rhythm or during coronary sinus pacing with a multi-electrode mapping catheter (HD Grid, Abbott, United States). During the ablation procedure, a contact force sensing, irrigated catheter, TactiCath SE (Abbott, United States) was used to deliver radiofrequency energy at powers of 30 to 50 W for 5–30 s. Pulmonary vein isolation was performed to achieve bidirectional block using wide antral circumferential isolation. Repeat electroanatomic mapping confirmed elimination of electrograms at the site of ablation lesions including elimination of fractionated signals. Non-pulmonary vein triggers were identified using isoproterenol infusion up to 30 mcg/min. Patients were excluded if any non-pulmonary vein triggers were identified. The effect on intraprocedural heart rate was determined based on change from the last sinus rate prior to initiation of ablation to the first sinus rate after ablation without the effect of isoproterenol or other chronotropic agents. Patients were clinically followed for recurrent arrhythmia with 3, 6, and 12-month extended monitors in addition to twice daily pulse checks and periodic and symptom drive ECG screening. Recurrent AF was considered to include any atrial arrhythmia including atrial flutter or atrial tachycardia.

Retrospectively, after completion of the procedure, a fractionation map was generated using a combination of parameters including a 10-ms width, a 30-ms refractory time, a 0.1 mV roving sensitivity, and a fractionation threshold of 2 [9]. Fractionation maps were generated without knowledge of the PVI lesion set. Areas of increased fractionation above the threshold were marked with white dots on the electroanatomic map. We associated areas of fractionation to six anatomic regions commonly associated with GP (left superior, left inferior, right superior, right inferior, posteromedial, and Marshall tract) and considered these areas potential GP sites. Fractionated signals outside of these six anatomic regions were excluded from the analysis. After the fractionation map was made and the potential GP regions were defined, the ablation lesions from the previously completed procedure were added to the map. The proportion of fractionated GP electrograms covered by ablation lesions was then calculated.

### Statistical analysis

Categorical variables are reported as counts with percentages. Continuous variables are expressed as means ± standard deviations or medians and interquartile ranges (IQR). Intraprocedural changes were compared using paired *t*-tests. Correlation between intraprocedural heart rate change and percent of fractionation ablated was performed using non-parametric tests by calculating the Spearman correlation coefficient (*ρ*). All statistical tests were 2-sided, with *p* < 0.05 indicating significance. Analyses were performed using Stata, version 14.1 (StataCorp, College Station, TX).

## Results

Of the 52 patients meeting inclusion criteria, the median age was 65 years (IQR 46–74), 42 (81%) were male, 37 (69%) had paroxysmal AF, and 15 (31%) had persistent AF (Table 1). Isolation of the pulmonary veins was achieved in all patients with confirmation of entrance and exit block. No non-pulmonary vein triggers were identified with provocative testing with isoproterenol. No additional ablation outside of the pulmonary vein isolation was performed in any patient. Left atrial maps included 2429 ± 821 points recording in sinus rhythm or during pacing from the coronary sinus using the multipolar mapping catheter. Patients were followed for a mean of 15.9 ± 5.2 with 13 (25%) having recurrent atrial arrhythmias after a 3-month blanking period. Organized atrial arrhythmias (atrial flutter or atrial tachycardia) were noted in 2 patients with the remainder of patients having only recurrent AF. Patients who had recurrent AF were more likely to have a history of persistent AF (62 vs. 18%, *p* = 0.03, Table 1).

**Table 1:** Patient characteristics at the time of atrial fibrillation ablation. *p* values are reported comparing patients with and without recurrent atrial fibrillation during the follow-up period. *p* values < 0.05 are considered significant and are noted bold

	All patients *n* = 52	Recurrent atrial fibrillation (*n* = 13)	Freedom from recurrent atrial fibrillation (*n* = 39)	*p* value
Age (years)	65 (IQR 46–84)	66 (IQR 59–84)	65 (46–75)	0.8
Male	42 (81%)	10 (77%)	32 (82%)	0.7
Type of atrial fibrillation				**0.003**
Paroxysmal	37 (71%)	5 (38%)	32 (82%)	
Persistent	15 (29%)	8 (62%)	7 (18%)	
Diabetes mellitus	6 (12%)	3 (23%)	3 (8%)	0.1
Obstructive sleep apnea	13 (25%)	5 (54%)	8 (15%)	0.2
Chronic kidney disease	3 (6%)	2 (15%)	1 (3%)	0.09
Coronary artery disease	3 (6%)	2 (15%)	1 (3%)	0.09
Left ventricular ejection fraction < 50%	7 (13%)	2 (15%)	5 (13%)	0.8
Beta-blocker use	60 (100%)	13 (100%)	39 (100%)	-
Anti-arrhythmic use	30 (58%)	10	20	0.1

Fractionated regions meeting the prespecified criteria and corresponding to areas of anticipated GPs were identified in all patients. The mean number of left atrial fractionated points identified per patient was 226 ± 124 with 71 ± 49 falling with the regions of anticipated GP. Of those 71 fractionation points found in anticipated GP regions per patient, a mean of 4 ± 6 was found in areas of low voltage (< 0.5 mV).

**Fig. 1:**
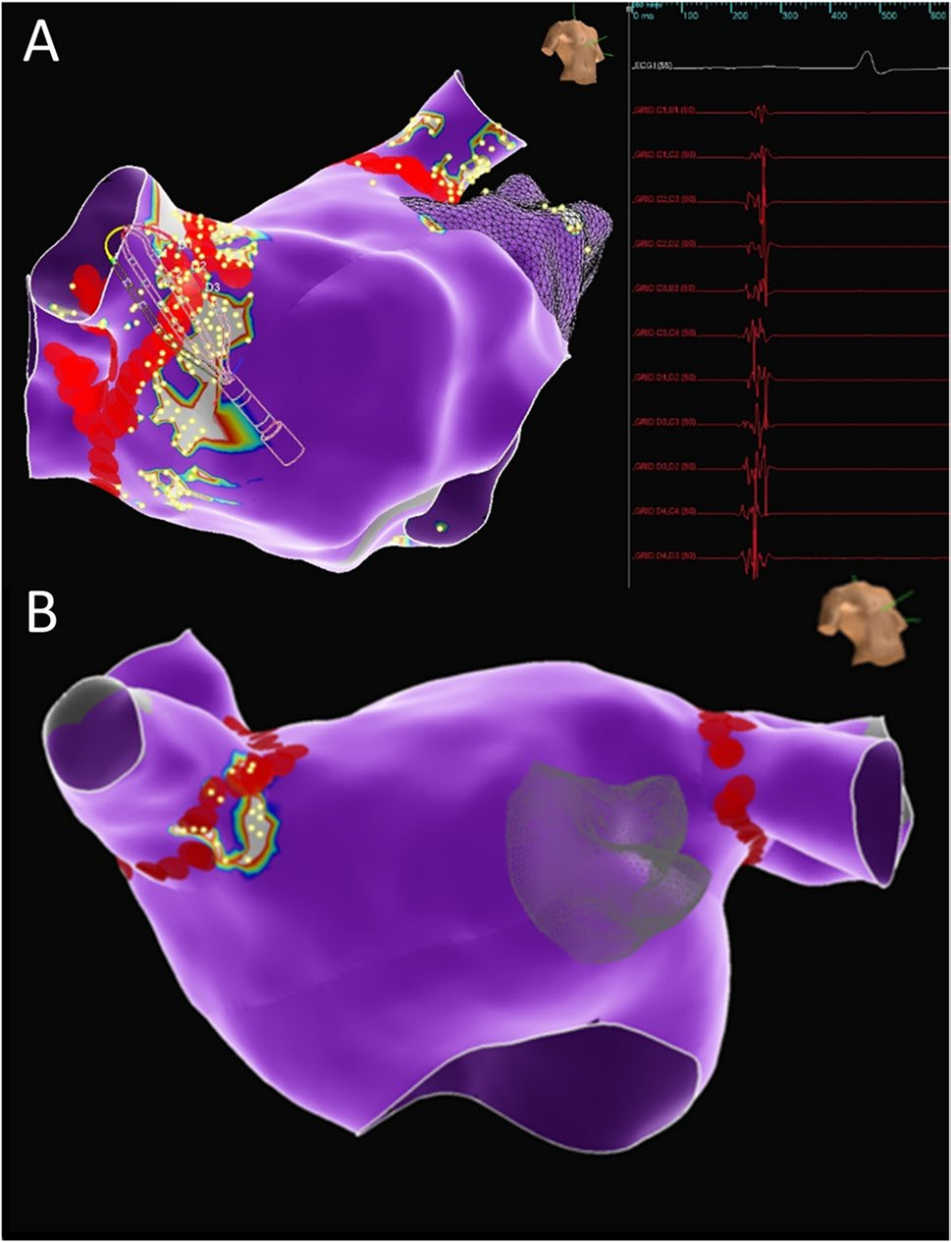
Examples of fractionated signals in areas corresponding to the right superior ganglionated plexus (GP) in two different patients. Yellow dots = electrograms meeting criteria for fractionation. Red dots = ablation lesion as part of pulmonary vein isolation. **A** Multipolar mapping catheter positioned in the region of the right superior GP with fractionated electrograms recorded in this location. There is a relatively large region of fractionation noted and only partially ablated by the pulmonary vein isolation ablation lesions. **B** A relatively smaller region of fractionation is noted in the region of the right superior ganglionated plexus in another patient. A larger, but still incomplete, percent of this region is ablated by the pulmonary vein isolation lesions

The most common sites of fractionation were identified in the region of the right superior and left superior GP (96% and 98%, respectively) with the right inferior GP being the least common to have associated fractionation identified (42%). There was substantial variation in the number of fractionated points noted at each GP, with the largest range observed at the right superior GP (range 3–120 points, Fig. 1 and Table 2).

**Table 2:** Fractionation noted and ablated within regions of anticipated ganglionated plexus

Ganglionated plexus region	Number of patients with fractionated signals in corresponding area (%)	Average number of fractionated points per region (range)	Percent of fractionated signals ablated per patient (SD)
Left superior	50 (96%)	17 (0–88)	36 (24)
Left inferior	25 (48%)	6 (0–55)	18 (20)
Marshall tract	33 (63%)	9 (0–46)	24 (21)
Right superior	51 (98%)	25 (3–120)	31 (21)
Right inferior	22 (42%)	4 (0–59)	10 (16)
Posteromedial	33 (63%)	4 (0–25)	0

**Fig. 2:**
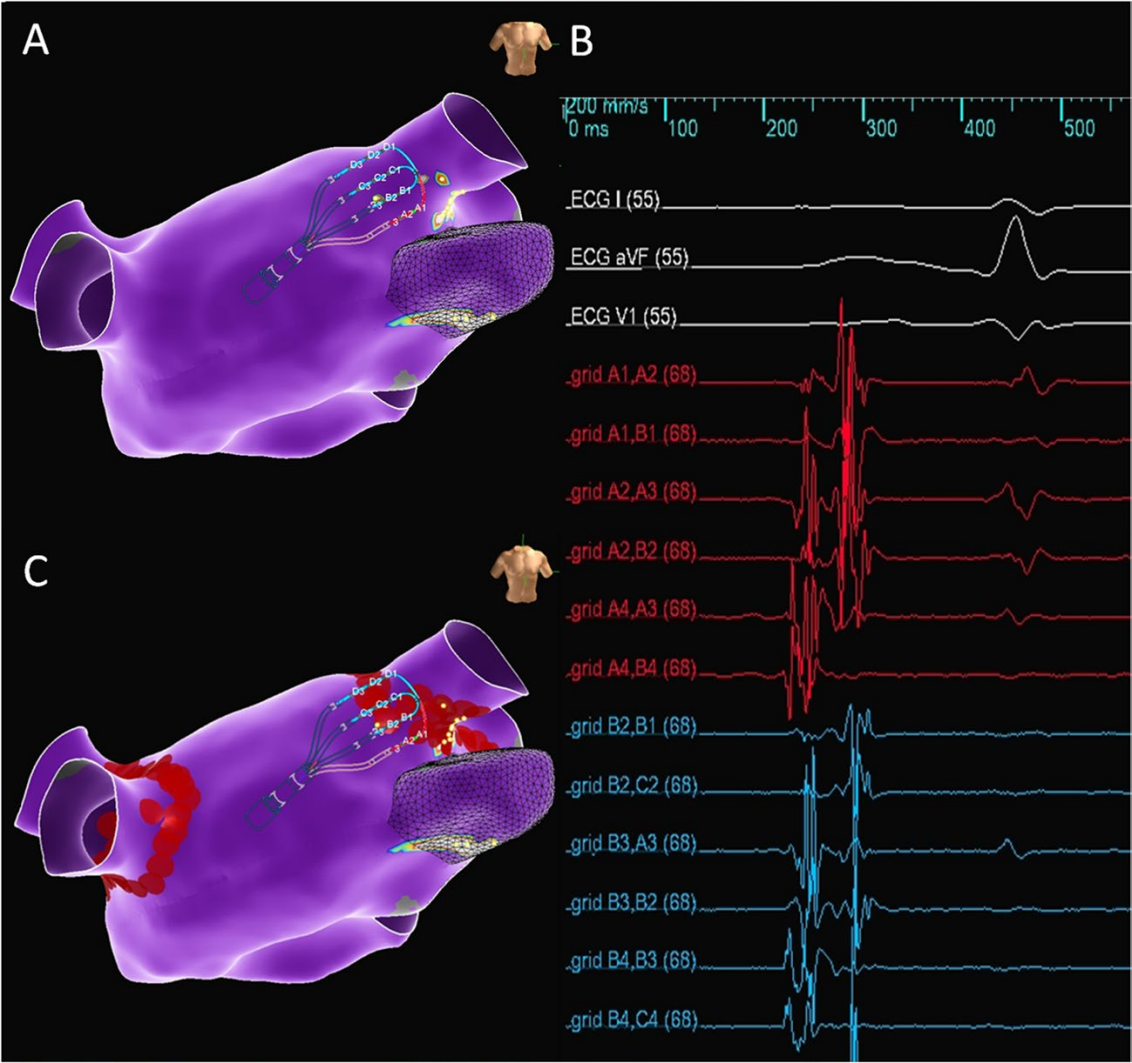
Example of fractionated signals in areas corresponding to the left superior ganglionated plexus (GP). Yellow dots = electrograms meeting criteria for fractionation. Red dots = ablation lesion as part of pulmonary vein isolation. **A** Multipolar mapping catheter positioned in the region of the left superior ganglionated plexus records fractionated electrograms (**B**). **C** Pulmonary vein isolation lesions in this region cover the area of fractionation

**Fig. 3:**
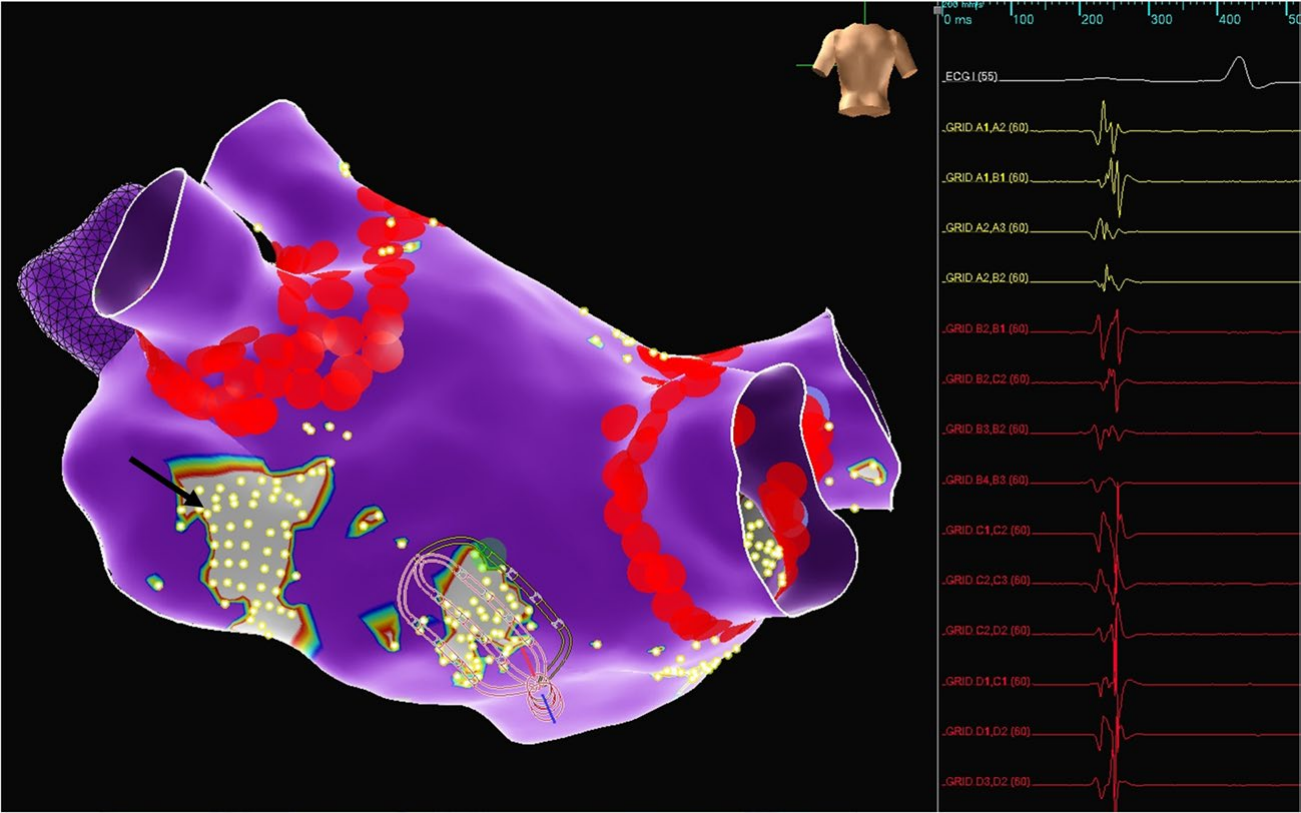
Example of fractionated signals in areas corresponding to posteromedial ganglionated plexus (GP) on the right (multipolar mapping catheter) and left inferior GP (arrow) region on, neither of which were ablated by the pulmonary vein isolation lesion set in this patient. The fractionated electrograms on the multipolar mapping catheter at the site of the anticipated posteromedial GP are shown on the right

Routine PVI wide antral circumferential ablation lesion set incidentally overlapped with fractionation in 50 (96%) patients. For the two patients without any incidental fractionation ablation, the region corresponding to the right superior GP was noted to be more ostial toward the pulmonary vein and covered by region of phrenic nerve capture. As a result, ablation was performed with a wider lesion set in this region, sparing region of likely GP innervation. On average, 26% of the fractionated electrograms corresponding with GP locations were incidentally ablated with this lesion set (Fig. 2). The proportion of areas of fractionated signals that were ablated differed significantly by area, with the highest proportion of fractionated areas ablated in the left superior (36%) and right superior (31%) GP regions (*p* = 0.01). Fractionated signals in the area corresponding to posteromedial GP were not ablated in any patient, although fractionated signals were identified in 33 (63%) patients (Fig. 3 and Table 2).

**Fig. 4:**
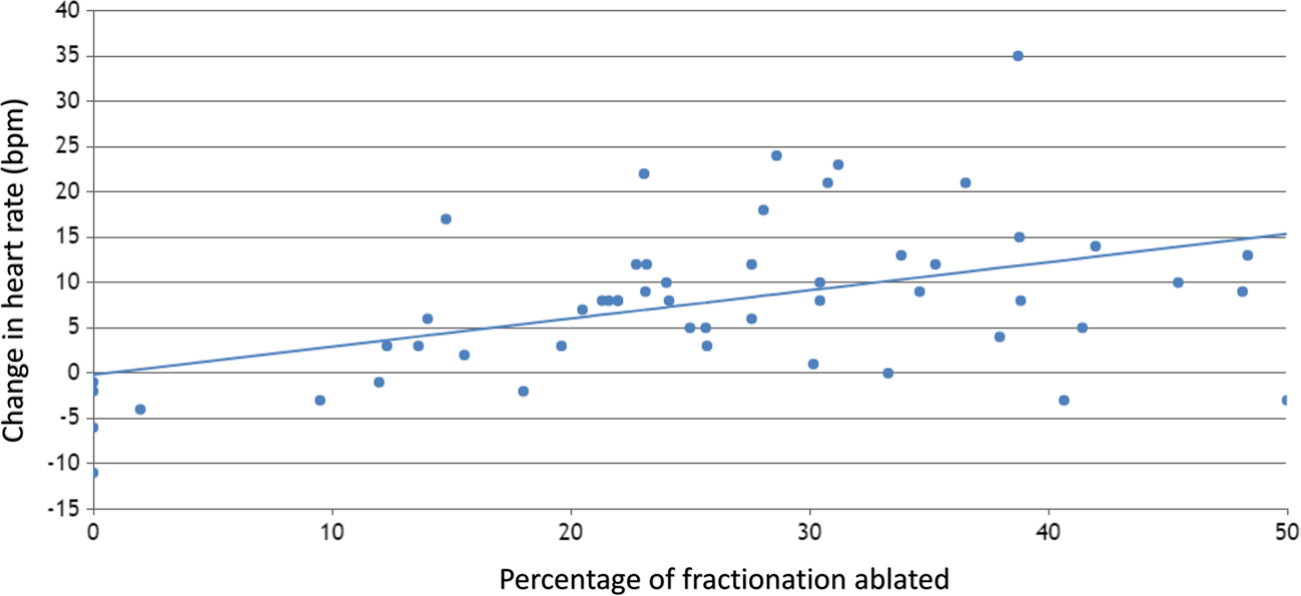
Scatterplot demonstrating the relationship between the percentage of fractionation in regions of anticipated ganglionated plexus that was ablated during routine pulmonary vein isolation and the change in intraprocedural heart rate. Spearman’s *ρ* = 0.5 (*p* < 0.001)

Intraprocedural heart rates prior to initiation of ablation increased from 63 ± 12 bpm to 71 ± 15 bpm (*p* < 0.001) after ablation. There was a significant association between the amount of fractionation in regions of anticipated GP that was ablated and the increase in intraprocedural heart rate *ρ* = 0.5, *p* < 0.001 (Fig. 4). There was no significant association between the number of ablation lesions performed and the increase in intraprocedural heart rate. There was no significant association between the amount of fractionation in the regions of anticipated GPs and subsequent recurrent AF. Patients with freedom from AF had a greater intraprocedural heart rate increase compared to those with recurrent AF (13 bpm vs. 1 bpm, *p* = 0.001).

## Discussion

During electrophysiological studies, various methods can be used to locate epicardial atrial GP that plays an important role in the regulation of cardiac function and in arrhythmogenesis. While there is no consensus on the most effective technique for locating GPs during electrophysiological studies, all available techniques have limitations. These methods include high-frequency stimulation (HFS), anatomic approach with or without imaging guidance, and fractionation mapping. HFS involves delivering electrical impulses at a high frequency to the myocardium to stimulate GP and observe their response, which can result in bradycardia and heart block as a result of parasympathetic activation [1, 5]. HFS is limited by lack of sensitivity, frequent induction of AF, either via direct atrial capture or by GP-induced AF trigger, and by the significant time required to perform HFS systematically. The anatomic-guided approach involves applying applications of radiofrequency energy over areas that are suspected to contain GP [10]. While it has been shown to be an effective approach in some studies, the results have been inconsistent and seem to result in higher rates of post-ablation macro re-entrant atrial tachycardia [11–13]. Due to the significant anatomic variability in GP size and location as well as various GP nomenclatures, it is not surprising that this approach would have significant limitations. We have also described using imaging guidance with pre-operative CT to identify epicardial adipose tissue, which correlates to areas of fractionation and GPs [14–16]. This technique is feasible and importantly demonstrates the significant variability in size and location of epicardial fat pads in individuals, potentially reflecting the limitations of a purely anatomically based approach. Understanding GP locations and the significance of incidental GP ablation during PVI is of increasing importance given the adoption of pulse field ablation, which does not cause durable denervation in the same fashion as thermal ablation approaches.

Despite the limitations in all forms of GP localization, electrogram characterization shows promise as a non-invasive, efficient, and accurate tool for identifying GP at the time of ablation based on the presence of multiple signals with varying amplitude, duration, and morphology within a single electrogram. While the association between GP and fractionation has been well described, the true mechanism for this has not been definitely established. It has been suggested that the presence of a high density of autonomic neural fibers within the atrial walls alters the normally homogenous cell-to-cell electrical conduction, resulting in poorly connected cells and fibrillary conduction through these regions [17]. Additionally, the GPs are thought to contain regions of high electrical resistance, which may also contribute to signal fractionation [18, 19]. Fragmented electrograms during sinus rhythm have been shown to predict vagal response during radiofrequency ablation [20]. The strategy of identifying fractionation as a marker of GP may benefit by the use of automated fractionation mapping software as evaluated in this study. With the establishment of objective criteria, this technique could improve the reproducibility of GP localization and therefore cardioneuroablation both for AF as well as other indications such as vasovagal syncope.

This study demonstrates that fractionation routinely identifies regions suggestive of GPs within the left atrium and that a degree of incidental cardioneuroablation is common during routine PVI. The acute increase in heart rate correlation with the degree of fractionation ablated supports the concepts that fractionation represents true GP and that more complete targeting of this fractionation may result in more complete denervation. Additionally, patients with freedom from AF during > 1 year of follow-up had a greater intra-procedural heart rate increase. This reinforces the significance of fractionation as a marker of atrial autonomic innervation and highlights the potential role of using fractionation to guide PVI to optimize denervation by guiding design of the lesion set. This partial cardioneuroablation may contribute to the effectiveness of AF ablation by preventing GP-associated triggers of AF, especially when those triggers may originate from extra-pulmonary vein sources, or potentially by modifying the heterogeneous conduction that creates substrate to maintain AF. Although physiologic evidence of cardioneuroablation has been associated with improved AF outcomes [21], further evaluation is necessary to demine whether adjusting PVI wide antral circumferential ablation lesion sets to target fractionated regions, leading to a more thorough cardioneuroablation, would improve AF outcomes.

### Limitations

The current study has limitations typical of a retrospective series. As cardioneuroablation was not planned in any case, no prospective evaluation of regions of anticipated GP (e.g., HFS) was performed to confirm fractionation corresponded to true regions of GPs, and evaluation of denervation with atropine was not performed. All ablations were performed under general anesthesia, which may blunt the acute heart rate changes associated with parasympathetic denervation. Conclusions about the risk of recurrent arrhythmias are limited in this population due to the small sample size.

## Conclusion

Patients undergoing AF ablation universally have fractionated electrograms identified in regions of anticipated sites of GP. Partial ablation of these regions frequently occurs incidentally during standard PVI. Additional investigation is necessary to determine the significance of cardioneuroablation guided by fractionation and to assess whether adjusting lesion sets to account for fractionated signals in areas associated with GP could result in improved outcomes.
